# Living with AIDS in Uganda: a qualitative study of patients’ and families’ experiences following referral to hospice

**DOI:** 10.1186/s12904-015-0066-3

**Published:** 2015-11-28

**Authors:** Wesley Too, Michael Watson, Richard Harding, Jane Seymour

**Affiliations:** Kabarak University, School of Medicine and Health Sciences, P O Private Bag-20157 Kabarak, Nakuru, Kenya; School of Health Sciences, University of Nottingham, Queen’s Medical Centre, Derby Road, Nottingham, NG7 2UH UK; The Cicely Saunders Institute, Bessemer Road, Kings College, London, SE5 9PJ UK

**Keywords:** Palliative care, HIV/AIDS, Africa, Hospice, Qualitative, Stigma, Poverty

## Abstract

**Background:**

Globally, the majority of people with HIV/AIDS live in sub-Saharan Africa. While the increasing availability of antiretroviral therapy is improving the outlook for many, its effects are yet to reach all of those in need and patients still present with advanced disease. This paper reports findings from qualitative interviews with patients living with AIDS and their caregivers who were receiving palliative care from Hospice Africa Uganda (HAU). We aimed to understand what motivated patients and their families to seek formal healthcare, whether there were any barriers to help- seeking and how the help and support provided to them by HAU was perceived.

**Methods:**

We invited patients with AIDS and their relatives who were newly referred to HAU to participate in qualitative interviews. Patients and carers were interviewed in their homes approximately four weeks after the patient’s enrolment at HAU. Interviews were translated, transcribed and analysed using narrative and thematic approaches.

**Results:**

Interviews were completed with 22 patients (10 women and 12 men) and 20 family caregivers, nominated by patients. Interviews revealed the extent of suffering patients endured and the strain that family caregivers experienced before help was sought or accessed. Patients reported a wide range of severe physical symptoms. Patients and their relatives reported worries about the disclosure of the AIDS diagnosis and fear of stigma. Profound poverty framed all accounts. Poverty and stigma were, depending on the patient and family situation, both motivators and barriers to help seeking behaviour. Hospice services were perceived to provide essential relief of pain and symptoms, as well as providing rehabilitative support and a sense of caring. The hospice was perceived relieve utter destitution, although it was unable to meet all the expectations that patients had.

**Conclusion:**

Hospice care was highly valued and perceived to effectively manage problems such as pain and other symptoms and to provide rehabilitation. Participants noted a strong sense of being “cared for”. However, poverty and a sense of stigma were widespread. Further research is needed to understand how poverty and stigma can be effectively managed in hospice care for patients for advanced AIDS and their families.

## Background

Two-thirds of the approximately 40 million people living with HIV/AIDS worldwide live in sub-Saharan Africa [1] where health resources are limited and existing health services struggle to provide even the most basic health care. While the increasing availability of HAART in Sub Saharan Africa is improving the outlook for many people infected with HIV, its effects are yet to reach many of those in need and many patients still present with advanced disease [2]. Recent evidence suggests that problems persist alongside HAART and that palliative care is still required [3]. Quantitative studies of patients with AIDS under hospice care in East Africa have reported a high prevalence of pain, symptoms and psychological distress [4], spiritual distress [5], and poor quality of life [6]. Qualitative data have revealed needs for communication and information [7]. Although poverty is a fundamental underlying problem [4], the social needs of people living with the highly stigmatised diagnosis of AIDS who are accessing palliative care in Africa are not well understood. Over the last decade or so the World Health Organisation African initiative has recommended that the best way to manage the care of patients with HIV/ AIDS is to provide palliative care alongside efforts to provide active treatment and prevent disease progression [5]. Therefore, understanding “total care needs” is essential for palliative care provision in the context of AIDS hospice care in poor contexts.

Uganda was the first country in sub Saharan Africa to designate palliative care as an essential clinical service and to include palliative care both in the government’s Strategic Health Plan and its HIV/AIDS National Strategic Framework [6]. An estimated 1.2 million people in Uganda are living with HIV/AIDS, with 64,000 AIDS-related deaths in 2009 [7]. A survey of palliative care needs in Uganda has shown that patients’ and families’ needs span three main areas: the relief of pain and other symptoms, counselling and basic financial support including food aid [8]. Many patients and families are caught in a ‘medical poverty trap’ [9] that is characteristic of the situation of most people affected by serious and terminal illness sub Saharan Africa. This means that it is essential that palliative care providers attend to poverty as well as to clinical care [2, 10].

This paper reports findings from qualitative interviews with patients living with AIDS and their caregivers who were receiving palliative care from Hospice Africa Uganda (HAU). We aimed to understand what motivated patients and their families to seek formal healthcare, whether there were any barriers to help- seeking and how the help and support provided to them by HAU was perceived in relation to their total care needs.

## Methods

### Design

Cross sectional qualitative interviews were conducted with patients with AIDS and their nominated family carers as part of a larger mixed methods study [11].

### Setting

Patients and their carers attending HAU were interviewed in their homes. The study was conducted in the capital city of Uganda, Kampala, and its environs.

HAU is the model hospice for Hospice Africa, founded in 1992. It seeks to provide affordable and culturally appropriate palliative care for patients with cancer and / or HIV/AIDS. There are now three hospices in Uganda, under an umbrella organization based in Kampala from which we recruited patients. The model of service involves out-patient care and home-based care within a defined operational area. HAU works closely with local hospitals and other health care facilities to accept referrals of patients suffering from pain and symptoms. However, the main source of referral is self-referral. By 31^st^ March 2014, HAU had provided support to 23, 653 patients since its inception [12].

### Participants

Over a period of six weeks in 2009, patients with stage III and IV HIV who were newly referred to HAU were invited to participate in the study. To be eligible for study inclusion, patients had to be at least 18 years old, living within six miles of HAU, and able to communicate in either English or Luganda. Patients were invited to nominate family member(s) closely involved in their care, so that these individuals could also be invited to take part in the study.

### Recruitment procedure

An information poster was displayed in the waiting area of HAU to facilitate patients’ general understanding of the project. Hospice staff identified potential participants when they enrolled at the service and, with the patient’s permission, referred them to the study researcher (WT) who provided then with verbal and written information. Written consent was then gained for WT to visit the patient at home, alongside the hospice outreach team. Separate consent was gained at the time of the home visit for the conduct of an interview. Family caregivers were similarly given information about the study, with patients asked to pass on a letter of invitation where this was required. Where nominated family members were in attendance at the time of the patient’s referral to the hospice, they were invited to participate in the study directly.

### Data collection

Interviews took place approximately 4 weeks after the patient’s enrolment at HAU. Patients and carers were interviewed together or singly, depending on their choice. Patients without a family carer were interviewed alone. An interview guide was designed in the light of existing literature. The guide sought to enable patients and/ or their caregivers to reflect on:

- Their experiences from when they first noticed they have HIV/AIDS
- What made them seek medical help
- Their experiences of the palliative care services received from HAU
- Their experiences of giving and receiving care and the difficulties and challenges they encountered.

Interviews lasted between 25 and 45 min. They were digitally-recorded. A bilingual assistant acted as a translator and also interpreter.

### Analysis

Where necessary, interviews were translated verbatim into English with the help of the bi-lingual assistant. Narrative summaries of each interview were written up to understand the context and content of each person’s or dyad’s experience and how their story evolved over time. This was followed by thematic analysis to compare experiences across all interviewees and identify commonalities [13]. The analysis was undertaken by WT without the use of a software package.

### Ethical committee review

The study received ethical committee approval from the Ugandan National Commission for Science and Technology (UNCST HS-508) and from HAU. The study proposal also received a positive review by the Research Ethics Committee in the Faculty of Medicine and Health Sciences, at the University of Nottingham [14] . Written consent was obtained for participants to be interviewed. Anonymity and confidentiality issues were addressed including use of pseudonyms names for participants.

## Results

Thirty-six patients were invited to participate in the study, of whom 30 agreed to a home visit for the purpose of undertaking an interview. Those who participated were very seriously or terminally ill. Many were bedridden and eight of patients died before being able to participate in an interview. Thus, 22 patients (10 female and 12 male) were interviewed. Participants’ age ranged from 18 to 60 years. Twenty family caregivers were nominated by patients to participate in the study and took part in joint interviews with the patient who nominated them. Socio-demographic and clinical profile of participants are described in Table 1.

**Table 1:** Socio-demographic characteristics and other clinical profile of participants

Socio-demographics characteristics [Baseline]		*N* = 22	%
Age	<20	1	4.5
	21–30	4	18.2
	31–40	6	27.3
	41–50	10	45.5
	>51	1	4.5
Gender	Male	12	54.5
	Female	10	45.5
Carer’s gender	Male	3	13.6
	Female	19	86.4
Carer’s relationship	Spouse	8	36.8
	Family member (sibling or relative)	14	63.6
Marital status	Never married/single	8	36.4
	Married	12	54.5
	Divorced/ widowed	2	9.1
Care setting of participants	Home care	11	50.1
	Out-patient	8	13.6
	In-patient	3	36.3
Residence status	Urban	8	36.3
	Semi-urban	6	27.4
	Rural	8	36.3
Clinical characteristics	Stage III	5	22.7
	Stage IV	17	77.3
Presence of co-morbidity	AIDS related cancers	18	81.8
	Opportunistic infections	4	18.2
Receiving Highly Active Antiretroviral Therapy (HAART)	Yes	19	86.4
	No	3	13.6

What prompted patients to seek help?

### Physical needs

Patients described persistent and troublesome symptoms as the main impetus behind their decision to seek help. However extreme poverty and worries about the financial implications of accessing medical care meant that most patients only sought this at a very late stage of their disease:

*We are struggling with many needs so we gamble our way to hospital…To move to get treatment is a problem. Body also is weak and this part (pointing at body) is painful and I still cough (Participant F)*

*Now I don’t have any money…just borrowing…not improving…feel a lot of pain. I feel like stealing money to pay for drugs…I wish I could die. (Participant E)*

Some patients described first going to informal health care before eventually seeking help from more formal sector. For example, the newly expanding availability of Chinese health care featured in some of the interviews; in others, reference was made to advice received from traditional African witchdoctors:

*So I went to a Chinese clinic …that is where I was buying that drug from which was very expensive but it didn’t help me…..I experienced skin problems that were not disappearing and I went to Dr. X’s clinic (a private oncologist). I also went to the Infectious Disease Institute to get HAART which has helped me to live and now [the] hospice doctor. (Participant E)*

*I have gone to a witchdoctor …it was my daughters who took me there and the witchdoctor would give us a pounded form of herbal medicines…with instructions to boil then and give me plenty to drink of it. (Participant D)*

Others resorted to drastic ‘self-help’ measures before seeking medical help. For example, participant C with Kaposi’s sarcoma reported how he had begun to try to cut the ‘blister’ from his arm when his neighbours and friends started to avoid him:

*People began fearing me when the wound opened up and the flesh started coming out…I did not know what to do with it, so I began to chop off the blistering growth in my arm. (Participant C)*

Patients were often almost completely incapacitated before any formal health care was sought. Overwhelmingly severe pain, fatigue, cough and dyspnea were common experiences:

*The wounds in the feet are disturbing me. By the time I got off the bed I had some little strength but I was breathing badly, and I said ‘Oh God, why am I breathing so badly’? (Participant B)*

*Just weakness and pain… the weakness is worrying her more because there are some things she would like to do on her own, naturally she is very hard working. (Daughter, caring for participant A).*

### Psychosocial and emotional distress

As well as urgent physical needs, distress caused by the psychosocial and emotional consequences of living with HIV/AIDS frequently lay behind patients’ and carers’ decisions to seek help and to eventually accept referral to hospice care. The strains of family care-giving and the intersection of emotional distress with poverty were particularly important. While poverty sometimes acted as a brake on help seeking behavior because of worries about additional financial burdens, the difficulties of providing family care in a situation where competing obligations became impossible to manage often prompted a search for help on behalf of the patient by their close family members:

*You know I take care of him but I have my family and children also to take care of. It is not easy but we try. I know I will have to take care of his children and mine at the same time in school [meaning currently and when the patient dies]. I count like I have two families to take care. It is money draining but what can you do? Now he has no energy/strength; he used to be truck driver. (A family caregiver, caring for his brother participant E).*

*You see we are struggling…the patient is lying there on the mat, no money for medication for the leg, no food, no drink; we are just there. (Participant G)*

Many family carers provided care in the context where they themselves were also ill or worried about their status: 11 patients also had carers who had HIV/ AIDS at various stages. Carers’ and patients’ worries about each other’s health and what would happen in the future were aggravated by a lack of access to the most basic resources:

*You see I am like this: she has diarrhoea, sometimes her clothes are soaking wet and I need to clean, I need gloves…I don’t even know if I have contracted [it], you see I don’t know my status. (Daughter-in-law of participant D)*

## What barriers were there to help seeking?

We have already alluded above to the dual role that poverty played in hindering and facilitating help seeking behaviour. Stigma was another key factor that played an important but complex role in relation to help seeking behaviour, acting both as a prompt and a key barrier to help-seeking.

### Stigma

Concerns about stigma were especially evident in the accounts of 15 patients and their family carers: they reported reluctance to disclose their disease status because of fears of losing: job security, their friends and any help that family and friends were currently providing. Patients weighed a number of issues before disclosing, including where they were living and who surrounded them. Where patients anticipated that they would receive sympathetic support from relatives or friends, they would disclose their status to lever access this support, which sometimes in turn enabled help to be sought from formal health care channels. In contrast, where participants felt that either there was no possibility of support or that disclosure would damage their relationships with others and undermine any help that they may already be receiving, they kept their diagnosis a secret. For example participant H, describes her selective disclosure to one relative but not to others, for fear of being discriminated against:

*Where I used to stay at my auntie’s place- I didn’t tell anyone of my status. The only person who knew was my brother. Where l live now at my brother’s house they know because they love me and no one discriminates (against) me; they treat me like any other family member (Participant H)*

In some cases where fears of stigmatisation had become a reality, this prompted a search for alternative forms of help out of sheer necessity and because of abandonment:

*When people at home discovered that participant F has HIV/AIDS and cancer they left us and abandoned us, so we learnt to be on our own….Now [our] money is over and we are now walking in the Kampala Street, hoping to get someone to help us. If we get a ‘good Samaritan’ we will thank God (Wife of Participant F)*

*I did not want people to see me…Even people at home started to isolate me… you find they say don’t use our basin, don’t use our cup… use your own cup… get your own plate ….I used to work in an hotel and she (boss) feared that I would stick to her in the final stages of my disease so she kicked me off the hotel (Participant I)*

In one case included in this study a comparatively young widow aged 39, had been abandoned by her husband’s family because of fears of contagion and had to return to her parental home. However, on her return, her immediate family struggled to care for her and she was put into an old, abandoned building where the only person in attendance was a young child. The child looked depressed and tired and was unable to attend school because of the duties thrust upon her. Participant B eventually accessed help from the hospice but died seven weeks after her referral, having been admitted to hospital from home for emergency intervention for dyspnoea.

## Experiences of services from Hospice Africa Uganda

All the patients who were interviewed described some form of positive impact from accessing hospice services. Immediate benefits included control of severe pain and other symptoms, with subsidiary benefits reported as relief of loneliness and a sense of being cared for. A contrast between hospice care and hospital care was sometimes drawn by patients:

*The wound [a fungating Kaposi’s sarcoma] was so painful, but now it is only ‘paralysed’ but not [so] painful. I used to experience lots of pain and did not have specialised care, but now I get visited like now you have come [to my] home. I am always lonely since my daughter is married and I am alone here (Participant J)*

*Even with all these drugs (showing them to the researcher) they have been giving me …the symptoms were not going. But since I came here and was given some treatment there is some improvement and I don’t have diarrhoea so much…you, the hospice, when I come to you, you take care of me and you give me some assistance. Because some hospitals you go to, no-one takes care of you and your illness gets worse (Participant K)*

Longer term rehabilitative impacts were also reported; these involved reactivation of social interaction or in some cases resumption of employment or schooling.

*There has been a big improvement, even people notice … I am able to interact with people now and move around. I can wear shoes…I feel good about myself now, since I am no longer smelling (Participant L)*

*I used to have … what I can I say it makes your face … depressed … like fear … but now I’m feeling better. You see, like now in the morning, I woke up and I had nothing to do … then I was trying to make a table in the workshop [place of employment] … Even when I go back to Kabale … I can dig … as you have found me working in the workshop and even go back to school. (Participant M)*

In some situations, participants reported that they had received support prior to their referral to HAU from several AIDS agencies that offered specialised services, but often at a cost. Patients were therefore particularly grateful for the free services they were offered at HAU. For instance, Participant N described how he was able to access morphine at no cost to himself when he was referred to HAU:

*I will tell you the situation I am in because before I was in a very bad situation but now I see a difference….In this illness, without telling lies, from the time I started going to the Infectious Disease Institute, I have not bought any medication [HAART].. I like hospice also for giving me the morphine for which I also don’t pay (Participant N)*

While most of the patients reported improvement in their physical condition when they enrolled for hospice care services, some perceived that in addition, HAU had helped them address their needs for subsistence. However, the hospice was limited in what it could provide meaning that patients’ expectations could not always be met:

*Last time you came you gave me some sugar and now it is finished and you know now I am not working and I am not leaving the house. I have backache and I have nowhere to get it. (Participant O)*

Some lived in hopes that the hospice would address all their needs, including provision of school fees for their children:

*‘Though I may want to work, I don’t have help. Any work I can do, for instance, before I was dealing in buying and selling of bananas but now there is no money. I want the hospice to assist my child, especially with school fees, because the father died and the child has no help. I don’t have money to take him to school (Participant P)*

## Discussion

This study explored the experiences of patients living with AIDS who were newly referred Hospice Africa Uganda and their caregivers. Qualitative interviews revealed the extent of suffering patients endured and the strain that family caregivers experienced before help was sought or accessed, and how poverty and stigma were, depending on the patient and family situation, both motivators and barriers to help seeking behaviour.

There are a number of limitations to the study conclusions. Our study involved a small sample of participants who were either self-referred or were referred by others to HAU. Caution should therefore be taken when considering the transferability of the findings. We relied on the HAU outreach team to identify and access patients’ homes and, as such, the researcher was looked upon by patients and their families as part of the home-visiting team. This may have introduced bias in the manner that participants spoke about their situation and may have deterred them from making any critical comments. An alternative approach may have been to return to interview participants without the hospice outreach team but this was impractical for reasons of travel costs and translator availability.

Our findings on the role of stigma in the lives of patients with AIDS resonate with a range of research undertaken in Uganda and other African countries [3, 13, 15], and particularly draw attention to the need for further research which examines the relationships between stigma, patients’ and families’ degree of emotional distress and their perceptions of, and access to, services. One study in South Africa [15] suggests that hospice care itself was experienced by AIDS patients and their families as stigmatising. This was not apparently the case in our study, possibly because of how well known and respected HAU was in the Kampala district where the study was conducted.

Our findings on the profound poverty of people with AIDS and their families lend further support to the need for palliative care in low income countries to be conceptualised more broadly than in high income countries [10], while the manner in which HAU was perceived to embrace the patient and their family as the unit of care arguably has lessons for how the provision of palliative care can be improved in high income countries.

Participants perceived that hospice care not only relieved their most urgent physical pain and symptoms (with some patients receiving morphine for the first time) but also in many cases enabled their participation in social life once more, thus supporting family care in the community.

## Conclusion

This research is the first study focused on patients’ and families’ experiences living with AIDS following referral to hospice in Uganda. Hospice care was highly valued and perceived effectively manage problems such as pain and other symptoms and to provide rehabilitation. However, poverty and a sense of stigma were widespread. Further research is needed to understand how poverty and stigma can be effectively managed in hospice care for patients for advanced AIDS and their families.

## References

[1] UNAIDS/WHO (2006). Overview of the global AIDS epidemic.

[2] Cox S, Gazzard B (2010). Models for palliative care in sub-Saharan Africa. Int Health.

[3] Lowther K, Harding R, Selman L, Higginson IJ. Experience of persistent psychological symptoms and perceived stigma among people with HIV on Antiretroviral therapy (ART)? A systematic review. Int J Nurs Studies*.* 2014, in press. 10.1016/j.ijnurstu.2014.01.015 24602830

[4] Grant E, Murray SA, Grant A, Brown J (2003). A good death in rural Kenya? Listening to Meru patients and their families talk about care needs at the end of life. J Palliat Care.

[5] Sepulveda C, Habiyambere V, Amandua J, Borok M, Kikule E, Mudanga B (2003). Quality care at the end of life in Africa. Br Med J.

[6] Ministry H (2010). Health sector strategic plan III 2010/11-2014/15.

[7] UNAIDS/WHO. UNAIDS report on global epidemic. In Global Report. 2010: Geneva; 2010.

[8] Kikule E (2003). A good death in Uganda: survey of needs for palliative care for terminally ill people in urban areas. Br Med J.

[9] Whitehead M, Dahlgren G, Evans T (2001). Equity and health sector reforms: can low-income countries escape the medical poverty trap?. Lancet.

[10] Clark D, Wright M, Hunt J, Lynch T (2007). Hospice and palliative care development in Africa: a multi-method review of services and experiences. J Pain Symptom Manag.

[11] Uren SA, Graham TM (2013). Subjective experiences of coping among caregivers in palliative care. Online J Issues Nurs.

[12] Hospice Africa Uganda Factsheet [http://www.hospiceafrica.or.ug/index.php/publications/fact-sheets/category/5-fact-sheets Accessed on 11th November 2015]

[13] Too W (2011). Palliative care for people living with HIV/AIDS in Uganda: an investigation of patients’ and caregivers’ experiences and professional perspectives.

[14] Governance: research ethics committee [http://www.nottingham.ac.uk/governance/universitycommittees/research-ethics.aspx accessed 31st July, 2015]

[15] Makhele MF, Mulaudzi FM (2012). The experiences of Batswana families regarding hospice care of AIDS patients in the Bophirima district, North West province, South Africa. Sahara J-Journal Social Aspects HIV/AIDS.

